# Intrinsic and Regulated Gonadotropin-Releasing Hormone Receptor Gene Transcription in Mammalian Pituitary Gonadotrophs

**DOI:** 10.3389/fendo.2017.00221

**Published:** 2017-09-04

**Authors:** Marija M. Janjic, Stanko S. Stojilkovic, Ivana Bjelobaba

**Affiliations:** ^1^Department of Neurobiology, Institute for Biological Research “Sinisa Stankovic”, University of Belgrade, Belgrade, Serbia; ^2^Section on Cellular Signaling, Eunice Kennedy Shiver National Institute of Child Health and Human Development, National Institutes of Health, Bethesda, MD, United States

**Keywords:** basal transcription, regulated transcription, gonadotrophs, gonadotropin-releasing hormone, gonadotropin-releasing hormone receptor

## Abstract

The hypothalamic decapeptide gonadotropin-releasing hormone (GnRH), acting *via* its receptors (GnRHRs) expressed in pituitary gonadotrophs, represents a critical molecule in control of reproductive functions in all vertebrate species. GnRH-activated receptors regulate synthesis of gonadotropins in a frequency-dependent manner. The number of GnRHRs on the plasma membrane determines the responsiveness of gonadotrophs to GnRH and varies in relation to age, sex, and physiological status. This is achieved by a complex control that operates at transcriptional, translational, and posttranslational levels. This review aims to overview the mechanisms of GnRHR gene (*Gnrhr*) transcription in mammalian gonadotrophs. In general, *Gnrhr* exhibits basal and regulated transcription activities. Basal *Gnrhr* transcription appears to be an intrinsic property of native and immortalized gonadotrophs that secures the presence of a sufficient number GnRHRs to preserve their functionality independently of the status of regulated transcription. On the other hand, regulated transcription modulates GnRHR expression during development, reproductive cycle, and aging. GnRH is crucial for regulated *Gnrhr* transcription in native gonadotrophs but is ineffective in immortalized gonadotrophs. In rat and mouse, both basal and GnRH-induced *Gnrhr* transcription rely primarily on the protein kinase C signaling pathway, with subsequent activation of mitogen-activated protein kinases. Continuous GnRH application, after a transient stimulation, shuts off regulated but not basal transcription, suggesting that different branches of this signaling pathway control transcription. Pituitary adenylate cyclase-activating polypeptide, but not activins, contributes to the regulated transcription utilizing the protein kinase A signaling pathway, whereas a mechanisms by which steroid hormones modulate *Gnrhr* transcription has not been well characterized.

## Introduction

The gonadotropin-releasing hormone (GnRH) receptor (GnRHR) is a member of a G protein-coupled receptor family (1). The receptor is expressed in pituitary gonadotrophs of all vertebrates, as well as in other tissues (2). The main signal transduction pathways of activated GnRHR in gonadotrophs is phospholipase C-β1-mediated phosphatidylinositol hydrolysis, thereby generating inositol-1,4,5-trisphosphate and diacylglycerol (3). Inositol-1,4,5-trisphosphate binds to its receptor at the endoplasmic reticulum membrane, leading to oscillatory Ca^2+^ release and Ca^2+^-dependent modulation of electrical activity (4). Diacylglycerol alone or together with Ca^2+^ activates protein kinase C (PKC) family of enzymes (5), whereas mitogen-activated protein kinases (MAPK) (6), phospholipase D (7), and phospholipase A2 (8) are PKC downstream signaling proteins. The coupling of GnRHRs to the synthesis of follicle-stimulating hormone (FSH) and synthesis and release of luteinizing hormone (LH) is critical for the establishment of hypothalamic–pituitary–gonadal axis, as these hormones regulate steroidogenesis and gametogenesis. In turn, gonadal hormones exhibit feedback effects at hypothalamic GnRH neurons and pituitary gonadotrophs (6).

The pituitary GnRHR number depends on developmental and reproductive stage and determines their responsiveness to GnRH. The receptor number is regulated, at least in part, at the transcriptional level (9). Cloning of GnRHR cDNA from numerous species facilitated investigations of GnRHR gene (*Gnrhr*) transcription. In general, transcription of the *Gnrhr* in gonadotrophs *in vitro* occurs in the absence (basal) and presence (regulated) of GnRH stimulation (2). The differences in the regulation of *Gnrhr* expression in mammalian species reflect differences in the promoter region of the gene (9, 10). The common aspect of regulated transcription of this gene is up- and downregulation by GnRH, depending on the pattern of application (11–13). Other hormones also contribute to regulation of *Gnrhr* transcription.

Here, we will mainly discuss *Gnrhr* transcription in the most frequently used mammalian models: rats, mice, sheep, and immortalized αT3-1 and LβT-2 gonadotrophs. We will first review the literature about *in vivo* GnRHR mRNA levels during development, aging and reproductive stage, followed by a brief description of rat and mouse *Gnrhr* structure and promoter region, basal vs. regulated activities, homologous upregulation of gene expression, and effects of gonadal and adrenal steroid hormones and other ligands on transcriptional activity of this gene.

## *In Vivo* Variations in *Gnrhr* Expression

**Figure 1:**
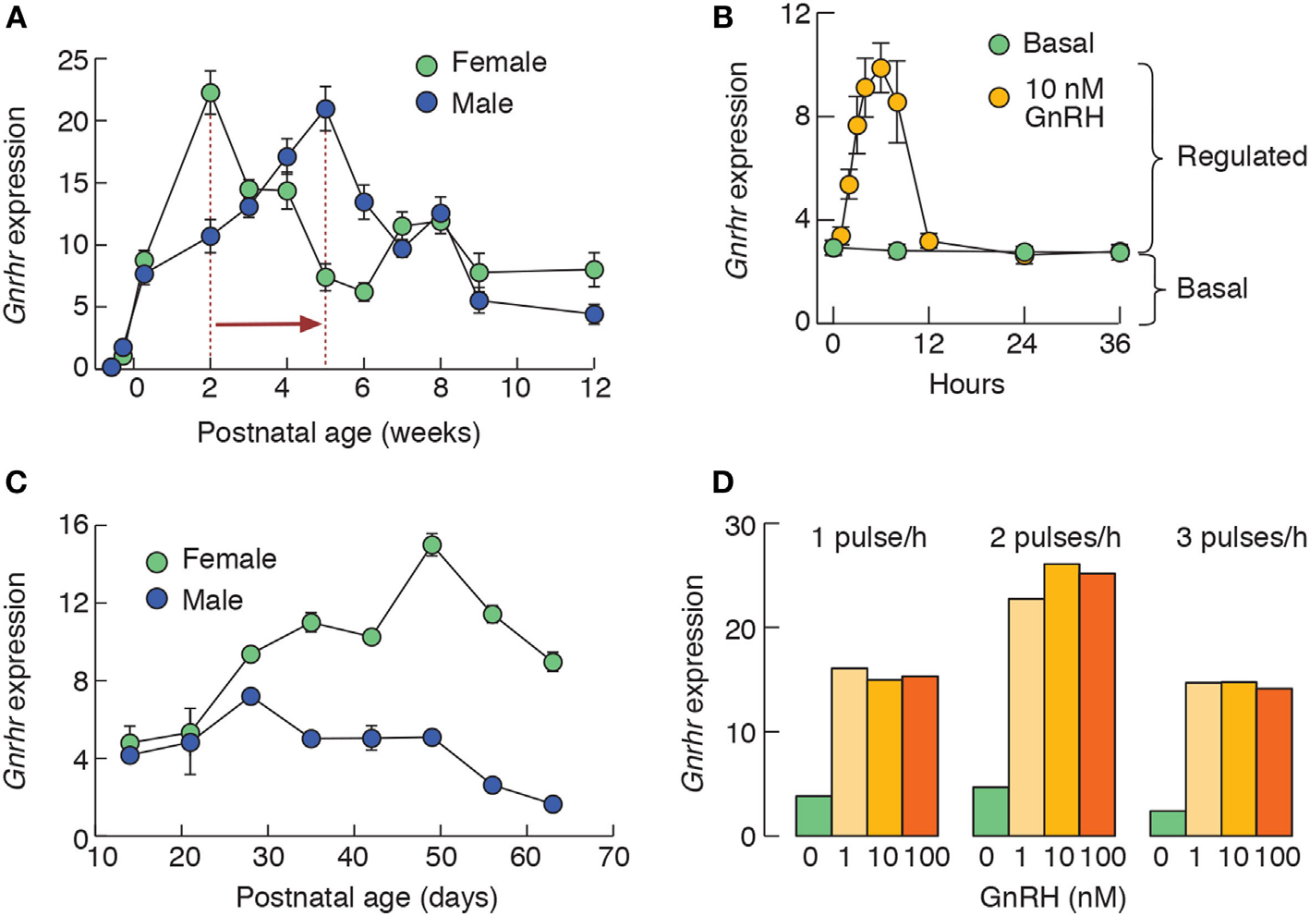
*In vivo* and *in vitro* expression patterns of rat pituitary *Gnrhr*. **(A)** Female and male developmental profiles of *Gnrhr* expression *in vivo*. Notice the differences in the peak of *Gnrhr* expression in females and males, as indicated by vertical dotted lines and a horizontal arrow. **(B)** Gonadotropin-releasing hormone (GnRH)-induced *Gnrhr* expression in 2-day-old static cultures of anterior pituitary cells from 7-week-old females. Cells were cultured in the absence or in continuous presence of 10 nM GnRH. Notice that desensitization of GnRH-induced *Gnrhr* expression does not affect basal expression. **(C)** The amplitude of GnRH-induced (10 nM continuously for 6 h) *Gnrhr* expression in female and male pituitary static cultures obtained from animals of different age is sex specific, in contrast to comparable levels of expression of this gene in both sexes *in vivo* **(A)**. **(D)** GnRH-induced *Gnrhr* expression in perifused pituitary cells from rat females. Cells were stimulated with 1, 10, or 100 nM GnRH for 1 × 5 min/hour, 2 × 5 min/hour, and 3 × 5 min/hour during 6 h. Notice that 1 nM GnRH was sufficient to induce maximum in response. This figure is derived from data published in Ref. (11, 16, 20); no permission is required from the copyright holder.

Developmental profile of *Gnrhr* expression in rats is depicted in Figure 1A. In females, *Gnrhr* expression increases rapidly over the first 2 weeks of development, followed by a transient decline and secondary rise in 7–8 weeks of age. In males, it increases gradually until 5 weeks of age (14–16), followed by a decline toward a steady expression at the adult age (11). The peak of *Gnrhr* expression during development correlates well with expression of gonadotropin subunit genes *Lhb, Fshb*, and *Cga* in both sexes (16) as well as with greater LH and FSH secretion in females, but not in males (17). These data are in accordance with the reports on GnRHR concentration and binding capacity during rat ontogeny (18, 19).

*Gnrhr* expression is downregulated in aged male rats (21), probably reflecting impaired GnRH secretion from the hypothalamus, because pituitary response to GnRH remains operative (22). However, in middle aged ovariectomized female rats, *Gnrhr* expression levels were lower than in young ovariectomized animals and the pituitary response to a steroid-induced gonadotropin surge was also impaired (23).

*Gnrhr* expression in the rat pituitary changes significantly during estrous cycle (24–26). Pituitary GnRHR mRNA content is relatively high on the mornings of diestrus I and diestrus II and declines sharply in the afternoons of diestrus days. However, higher *Gnrhr* expression can again be observed in the late evening of diestrus II (26). During proestrus, a sharp rise in *Gnrhr* expression occurs between morning and noon, followed by oscillation in expression until 17:00 h, when a second peak can be observed (25). It should also be noted that maximal binding of D-Ala^6^-GnRH, a synthetic GnRH analog, occurs at diestrus II as well, indicating that the maximal number of GnRHRs during the cycle is reached before proestrus (27). Estrous is characterized by low *Gnrhr* expression (24, 25). In general, the changes in pituitary *Gnrhr* levels correlate well with GnRH content and *Gnrh* expression in the hypothalamus (25, 26). In sheep, GnRHR mRNA expression and GnRH binding increase over the luteal phase and decline after the preovulatory LH surge, reaching the lowest levels 24 h after estrous (28–30).

Ovariectomy in rats and mice reduces the pituitary GnRHR numbers (31, 32), combined with marked upregulation of GnRHR mRNA (33). Interestingly, in ovariectomized rats, hypothalamic *Gnrh* and pituitary *Gnrhr* expression levels fluctuate during the day (26). In castrated male rats, there was a rise in mRNA, and receptor number, GnRHR affinity for GnRH, and gonadotropin secretion, which was, at least partly, prevented by a testosterone replacement therapy (33–38). Similarly, castration induces upregulation of *Gnrhr* expression in sheep (39). By contrast, castration was shown to induce a fall in mouse pituitary GnRHR numbers (40).

Rat pituitary responsiveness to GnRH remains low until 12th day after conception and then rises to reach maximum on the first day postpartum (41). We also noticed lower aplitude of GnRH-induced expression of dentin matrix protein 1 in gonadotrophs from pregnant female rats (20). These data imply that GnRHR mRNA content changes during pregnancy in rat, although this was not investigated. By contrast, pregnancy does not induce changes in GnRHR numbers or mRNA levels in sheep, suggesting that other mechanisms account for a fall in maternal pituitary responsiveness (42). Number of GnRHRs (43–45) as well as *Gnrhr* expression levels (46) are low during lactation in rat (probably reflecting diminished GnRH secretion from the hypothalamus), but rise rapidly after pup removal (45, 46).

## The Structure of *Gnrhr* Promoter Region

The 5′-flanking sequences of rat and mouse *Gnrhr* promoter have been isolated and characterized (47–50). In these species, *Gnrhr* gene is present as a single copy, positioned on chromosome 14 and 5, respectively, and contains three coding exons and two introns (10). Both promoters share strong homology over the region 1.2 kb upstream of the ATG codon (50). In this region, two identical response *cis*-elements of the mouse promoter are present in the rat *Gnrhr* promoter, a canonical activating protein 1 and steroidogenic factor 1 (also present in the ovine promoter; SF1 or NR5A1) (9). The rat promoter contains two additional response elements that are held responsible for functional differences between rat and mouse promoter: an imperfect cAMP response element, suspected to convey pituitary adenylate cyclase-activating peptide (PACAP) actions, and an element confined to −252/−245, that binds a protein yet unidentified, termed SF1 adjacent protein or SAP. All of these elements are required to mediate the gonadotroph-specific activity (51–53). An element termed the *Gnrhr* activating sequence, which could confer activin actions in mice, is also present in the rat promoter, but it is inactive (54). Comparing to the mouse promoter, where all known response elements fall in the proximal region, an additional regulatory region containing *Gnrhr*-specific bipartite enhancer (GnSE) is situated on a more distal part of the rat promoter. Thus, for the full gonadotroph-specific activity of the rat promoter, additional distal elements within the −1,150/−750 bp region are required. Two major response elements located at positions −994/−960 and −871/−862 are responsible for GnSE action (51, 52). Maximal GnSE activity requires the presence of SF1 response element located in the proximal domain. Both GnSE elements bind LIM-homeodomain proteins LHX3 and ISL1 and this seems to be crucial for gonadotroph-specific expression of the gene (9, 52, 55). For the detailed structure of rat and mouse promoters, see Ref. (9). The functional properties of the ovine *Gnrhr* promoter region were not investigated in details; however, the analysis of the 5′-UTR indicates that different mechanisms evolved for pituitary specific expression of *Gnrhr* in sheep and rodents (56).

## Basal and GnRH-Regulated *Gnrhr* Expression

Several lines of evidence indicate that *Gnrhr* expression is inherent to gonadotrophs. Some functional receptors must be present in gonadotrophs in Kallmann syndrome patients to explain how GnRH administration restores pituitary and gonadal functions (57). In agreement with this, *Gnrhr* expression is detectable and functional GnRHRs are present in the rat gonadotrophs *in vitro* even after prolonged period of GnRH absence (58). Furthermore, prolonged continuous GnRH application does not completely stop *Gnrhr* transcription (Figure 1B) (11). Finally, naïve (never stimulated) αT3-1 and LβT-2 cells express functional Ca^2+^-mobilizing GnRHRs (59, 60).

In rat, mouse, and sheep, the main positive regulator of *Gnrhr* transcription is GnRH itself (11–13, 61), depending on the pattern of GnRH application. Figure 1B illustrates that continuous stimulation of rat pituitary cells induces a transient induction of *Gnrhr* transcription, with maximal response at 6 h (11, 13). Longer GnRH stimulation leads to downregulation in *Gnrhr* transcription (11, 62). Therefore, it is reasonable to postulate that pulsatile GnRH stimulation is required not only for gonadotropin subunit expression, but also for the proper regulation of *Gnrhr* expression (63, 64). In the rat pituitary cells, 6 h application of GnRH in two pulses per hour, each lasting 5 min, provides the highest amplitude of response (Figure 1D). By contrast, immortalized gonadotrophs do not respond to GnRH application with upregulation in *Gnrhr* transcription (11, 65). This could reflect their embryonic origin or the side-effects of immortalization procedure. However, short GnRH stimulation increases GnRHR binding in αT3-1 membranes, without apparent effect on *Gnrhr* expression (66). Continuous GnRH application in αT3-1 also does not affect GnRHR mRNA levels, but downregulates GnRHR numbers (65). Thus, GnRHR signaling also engages translational regulation. Interestingly, GnRHR signaling induces remodeling of ribosome content in LβT-2 cells (67).

We also noticed that basal and GnRH-induced *Gnrhr* expression depends on the age and sex of rats used for pituitary cell preparation when cells are cultivated in the absence of steroid hormones. Although the relationship between basal and GnRH-stimulated transcriptional activity is comparable in both sexes, the amplitude of response to GnRH increases in female from juvenile to adult stage, but this is not the case with male rat cells (Figure 1C) (11). It is interesting to speculate that epigenetic modifications may have a role in the observed differences, although *Gnrhr* promoter regions in mouse and rat are not rich in cytosine–phosphate–guanine islands (68).

Gonadotropin-releasing hormone-induced *Gnrhr* expression relies, at least partially, on PKC activation and subsequent MAPK phosphorylation. The localization of the GnRHR in the lipid rafts (69) is important for activation of these signaling pathways (70, 71). The roles of different PKC isoforms in activation of the “classical” MAPK signaling pathways, composed of extracellular signal-regulated kinase (ERK1/2 and ERK5), c-Jun N-terminal kinases (JNK1/2) and p38, were characterized in immortalized gonadotrophs (72–75), but not in native gonadotrophs. MAPKs activate Fos and Jun proteins, which form a complex that binds to the AP1 site. GnRH itself also induces *Fos, Jun*, and *Junb* transcription in the rat gonadotrophs (20, 76). GnRH-induced *Gnrhr* expression in dispersed rat pituitary cells seems to depend mostly on ERK1/2 pathway, with a small but significant involvement of p38 and ERK5 (11). Intriguingly, although JNK1/2 was shown to play a critical role in GnRH induction of the *Gnrhr* expression in αT3-1 cells (77), inhibition of JNK1/2 had no effect on basal or GnRH-stimulated *Gnrhr* expression in the primary cultures (11). Whether this means that, in the rat gonadotrophs, Jun proteins are activated trough alternative pathways or that they are already active in a manner sufficient to induce transcription, remains to be elucidated.

Basal *Gnrhr* transcription also depends on PKC–MAPK signaling pathway (11). However, the existence of basal *Gnrhr* expression during continuous GnRH application could be explained by the fact that the signaling pathways downstream of PKC may also be activated by other factors, whose signaling converges to MAPKs. Indeed, increased Ca^2+^ influx, which in gonadotrophs is also stimulated by PKC (78), is sufficient to induce *Gnrhr* transcription (11), which may imply the possible role of calmodulin in activation of MAPKs (79). Also, portions of ERK1/2 and p38 are phosphorylated and therefore active under basal conditions in immortalized gonadotrophs (74, 80). Although infertile, female ERK1/2 knockout mice also retain *Gnrhr* expression in the pituitary (81), indicating that basal *Gnrhr* expression only partially relies on this pathway, at least in the mouse. Accordingly, cFos-deficient mice show an aberrant, but not completely abolished *Gnrhr* expression (82). In addition, in the rat pituitary cells, ERK inhibition cannot eliminate GnRH-induced *Gnrhr* transcription completely (11).

## Dependence of *Gnrhr* Expression on PACAP and Activins

Pituitary adenylate cyclase-activating peptide from hypothalamus may reach the pituitary, but could also be synthetized in the pituitary by gonadotrophs and folliculostellate cells (83), i.e., it could act as an autocrine/paracrine regulator of gonadotrophs by activating its PAC1 receptor expressed in these cells (84). Like GnRH, PACAP activates Ca^2+^ release in inositol-1,4,5-trisphosphate-depedent manner (85), but also increases cAMP production, leading to an activation of protein kinase A (86). A high pulse frequency PACAP administration to LβT-2 cells induced *Gnrhr* transcription (87) and in αT3-1 cells with a rat *Gnrhr* construct, dibutyryl-cAMP increased promoter activity (49). On the other hand, PKA stimulation by forskolin failed to induce *Gnrhr* transcription in LβT-2 cells (11). Although a bipartite element in the rat *Gnrhr* promoter was identified and termed as PACAP response elements I and II (53), the role of PACAP in regulation of *Gnrhr* expression in rat, mouse, and sheep gonadotrophs should be further investigated.

Activin-A stimulates GnRHR synthesis in pituitary cells from juvenile female rats. This effect could not be abolished by inhibin (88) and probably is posttranscriptional; unlike mouse, rat promoter region does not contain a functional activin response element (10). Although activin A, alone or in synergy with GnRH was shown to influence *Gnrhr* transcription upregulation in αT3-1 cells (89, 90), activin receptor II is not required for *Gnrhr* expression in mice (91). For more details on *in vitro* and *in vivo* actions of activins, see Ref. (92).

Prolonged inhibin treatment of the rat pituitary cells cuts the number of GnRHR in half (93), while in ovine pituitary cell culture, 48 h inhibin treatment increases GnRHR binding (94). Continuous microinfusion of inhibin downregulates GnRHR mRNA levels in immature male rats, but this effect could not be observed in adult animals (15).

## Dependence of *Gnrhr* Transcription on Steroid Hormones

In intact rats and sheep, serum estradiol correlates well with increased GnRHR numbers (27, 95), suggesting stimulatory effect of this steroid on transcriptional and/or posttranscriptional events. In contrast to estradiol, progesterone suppresses *Gnrhr* transcription and downregulates pituitary responsiveness to GnRH in mammals (94, 96–98). Progesterone treatment also reduces GnRHR mRNA levels after LH surge in estradiol primed ovariectomized female rats (96). Furthermore, it was suggested that a decrease in progesterone, rather than an increase in estradiol, during luteolysis is responsible for the increase in GnRHR mRNA and GnRHR number in the ovine pituitary (99–101). In male rats, there was a negative correlation between GnRHR-binding capacity and testosterone levels in serum (18, 19), further suggesting that androgen treatment also inhibits *Gnrhr* transcription/posttranscriptional events. However, these *in vivo* experiments could not dissociate between the direct effects of gonadal steroid hormones on *Gnrhr* transcription from the indirect effects mediated by modulation of GnRH secretory pattern. Gonadotrophs from castrated rats showed fewer GnRH-induced spike–plateau Ca^2+^ responses than cells obtained from intact rats (102), which could be reversed by treatment with a testosterone analog, thus implying its direct effect (102, 103).

The estradiol regulation of the GnRHR numbers in sheep was extensively studied [for review, see Ref. (104)]. *In vivo* administration of estradiol in orchidectomized sheep increased GnRHR mRNA content (105). Similarly, in ovine pituitary cultures, prolonged estradiol treatment increased the number of GnRHRs (106) and *Gnrhr* expression, which was greatly attenuated by progesterone (94, 107). Addition of progesterone alone also reduced GnRHR binding (94). By contrast, neither estradiol nor progesterone affect basal *Gnrhr* expression in the female rat pituitary cells, while progesterone inhibits GnRH-induced *Gnrhr* expression (108). In αT3-1 cells, estradiol reduced GnRHR numbers and mRNA (109, 110), but did not affect *Gnrhr* expression in LβT-2 cells (111).

However, an estradiol responsive element is not present within rat or mouse and ovine *Gnrhr* promoter (9, 10, 56) and rat *Gnrhr* promoter region does not contain the progesterone binding element (9, 10). It was suggested that estradiol effect on *Gnrhr* transcription occurs through membrane associated estrogen receptor-α (112), while mechanism(s) of progesterone action remain unclear.

It is well established that adrenal glucocorticoids affect reproduction (113), but the role of endogenous glucocorticoids in the regulation of *Gnrhr* expression in rats and mice has not been systematically investigated (114). However, continuous infusion of cortisol did not change *Gnrhr* expression in orchidectomized sheep, although it reduced the amplitude of estradiol-induced *Gnrhr* expression upregulation (115, 116). Studies in rats showed that corticosterone and cortisol do not have an effect on GnRHR numbers (117, 118). Dexametasone stimulated *Gnrhr* expression in LβT-2 cells (111, 119). In mouse *Gnrhr* promoter, an activating protein 1 containing site was identified as a mediator of dexamethasone induced transcription (120, 121).

## Conclusion

*Gnrhr* transcription is a functional marker of differentiated gonadotrophs. It occurs in the absence of any stimuli and is regulated by several hormones (Table 1). The main regulator of transcription of this gene is hypothalamic GnRH and pulsatile GnRH exposure is needed to sustain this process. Transcription is also facilitated by PACAP in an autocrine/paracrine manner, while activins are unlikely to play a physiological role in *Gnrhr* transcription. Steroid hormones influence *Gnrhr* transcription through regulation of GnRH secretion and directly, through a largely uncharacterized mechanisms. The mouse immortalized αT3-1 and LβT-2 cells remain, to this day, the best characterized gonadotroph cell model, although data obtained using these cells do not always correlate with findings in primary mouse and rat pituitary cells. Further studies are needed to elucidate signaling pathways accounting for control of *Gnrhr* transcription, especially in sheep. This includes the possible effects of gonadectomy or steroid hormone application on MAPK signaling.

**Table 1:** Up- and downregulation of *Gnrhr* expression by hypothalamic, intrapituitary, gonadal, adrenal hormones, and factors.

	Upregulation	Downregulation	No effect
Rats *in vivo*	GnRH, E2 (122)	P (96), Cetrorelix (123), Inhibin (15)	
Rat pituitary cells	GnRH (11, 20)		E2, P (108)
Mouse pituitary cells	GnRH (11)		
Mouse LβT-2 cells	Dexamethasone (111, 119), PACAP (87), Activin-A (119)		GnRH (11), E2 (111)
Mouse αT3-1 cells	GnRH (77), Triptorelin (110), Dibutyryl-cAMP (49), Activin-A (89)	E2, P (110)	GnRH (65)
Sheep *in vivo*	GnRH (61), E2 (62, 116, 124)	P (98)	Cortisol (115, 116)
Ovine pituitary cells	E2 (106)		

*Triptorelin, GnRHR agonist; Cetrorelix, GnRHR antagonist; E2, estradiol; P, progesterone; T, testosterone; PACAP, pituitary adenylate cyclase-activating polypeptide; GnRH, gonadotropin-releasing hormone*.

*Numbers in parentheses indicate the corresponding references*.

## Author Contributions

All authors (MJ, SS, and IB) contributed to the writing of the manuscript.

## Conflict of Interest Statement

The authors declare that the research was conducted in the absence of any commercial or financial relationships that could be construed as a potential conflict of interest.
